# Mycotoxins during the Processes of Nixtamalization and Tortilla Production

**DOI:** 10.3390/toxins11040227

**Published:** 2019-04-16

**Authors:** Sara Schaarschmidt, Carsten Fauhl-Hassek

**Affiliations:** German Federal Institute for Risk Assessment (BfR), Department Safety in the Food Chain, Max-Dohrn-Str. 8-10, D-10589 Berlin, Germany; carsten.fauhl-hassek@bfr.bund.de

**Keywords:** aflatoxins, alkaline, hydrolyzed fumonisins, fumonisins, food processing, maize, masa, matrix-associated mycotoxins, modified mycotoxins, tortillas

## Abstract

Tortillas are a traditional staple food in Mesoamerican cuisine, which have also become popular on a global level, e.g., for wraps or as snacks (tortilla chips). Traditional tortilla production includes alkaline cooking (nixtamalization) of maize kernels. This article summarizes the current knowledge on mycotoxin changes during the nixtamalization of maize and tortilla production. Upon nixtamalization, mycotoxins can be affected in different ways. On the one hand, the toxins can be physically removed during steeping and washing. On the other hand, mycotoxins might be degraded, modified, or released/bound in the matrix by high pH and/or high temperature. This also applies to the subsequent baking of tortillas. Many studies have shown reduced mycotoxin levels in alkali-cooked maize and in tortillas. Most of the available data relate to aflatoxins and fumonisins. The reduction (and detoxification) of aflatoxins during nixtamalization might, however, be partially reversed in acidic conditions. The loss of fumonisin concentrations is to some extent accompanied by hydrolyzation and by lower toxicity. However, some studies have indicated the potential formation of toxicologically relevant modified forms and matrix-associated fumonisins. More data are required to assess the influence of alkaline cooking regarding such modified forms, as well as mycotoxins other than aflatoxins/fumonisins.

## 1. Introduction

Mycotoxins are secondary fungal metabolites that are produced in the field and/or during the storage of crops and raise health concerns for humans and animals due to their toxic potential. Typically, several mycotoxins occur in parallel in crops. They can be produced by different fungal species, but single species are also usually capable of producing a distinct set of toxins [1]. Aflatoxins are mainly produced by *Aspergillus* and *Pencillium* species, with aflatoxin B1 (AFB1) being the most toxic and carcinogenic. Aflatoxins—including AFB1, aflatoxin B2 (AFB2), aflatoxin G1 (AFG1), and aflatoxin G2 (AFG2)—are particularly common in maize and other crops produced in warmer climates and are a serious health threat in many regions worldwide (for more information see e.g., [2,3]). Fumonisins belong to a large group of toxins referred to as *Fusarium* toxins that are produced by several *Fusarium* species, such as *Fusarium verticillioidies*. Moreover, fumonisins can be produced by some species of the *Aspergillus niger* complex. Fumonisins B1, B2, and B3 (FB1, FB2, FB3) are frequently found in raw maize and can exhibit liver and kidney toxicity [4]. Other *Fusarium* toxins, which are often present in maize, include zearalenone (ZEN [5]) and trichothecenes, such as deoxynivalenol (DON) [6,7]. In addition to such ‘traditional’ mycotoxins, which have been assessed and monitored in relative depth, so called ‘emerging’ mycotoxins have been identified. Similar to traditional mycotoxins, emerging mycotoxins are directly produced by fungi. Although some have been known for several decades already, emerging mycotoxins are still, however, less investigated and understood. One example of emerging mycotoxins is moniliformin (MON), which is also produced by some *Fusarium* species [8]. 

In addition to the free mycotoxins produced by toxigenic fungi (free parent compounds), mycotoxins can be modified in their chemical structure by biological or chemical processes [9,10,11]. Further, mycotoxins can be connected to the matrix, either by being physically entrapped or by covalent binding to matrix molecules. A proposal for a harmonized terminology of modified and matrix-associated mycotoxins was provided by Rychlik et al. [11]. Such forms can raise analytical challenges, which is particularly true for matrix-associated mycotoxins. For those, special treatments of the matrix, after extraction of free mycotoxins, are required to be able to extract the bound forms. However, free modified forms are, similar to emerging mycotoxins, often not covered by routine analysis. 

Food processing, in general, is capable of affecting mycotoxins present in the raw materials. A reduction in mycotoxin concentrations might be caused by fractionation or (partial) degradation of the toxins, leading also to lower toxicity. However, often, lower mycotoxin levels (also) involve modification or binding mechanisms. In such cases, the resulting structures might still harbor unknown toxicity or might be (re)converted into a toxic form. Further, concentrations of free toxins can even increase during food processing by releasing mycotoxins from matrix components (if matrix-associated mycotoxins are present in the raw material and depending on the processing conditions). Tortillas are a traditional staple food for the Mesoamerican population and are increasing in popularity throughout the world [12], which also applies to related products, such as tostadas, tortilla chips, and maize chips. Their unique flavor is caused by an alkaline cooking of maize—a special processing procedure called nixtamalization. This process can cause several physicochemical changes in maize kernels and is capable of affecting mycotoxins. This review presents the current knowledge on mycotoxin changes during the process of tortilla production with a particular focus on the nixtamalization step. In doing so, changes in the concentration of free parent forms are considered, as well as their potential modification and the putative toxicological impacts. 

## 2. Processes Involved in Nixtamalization and Tortilla Production

**Figure 1:**
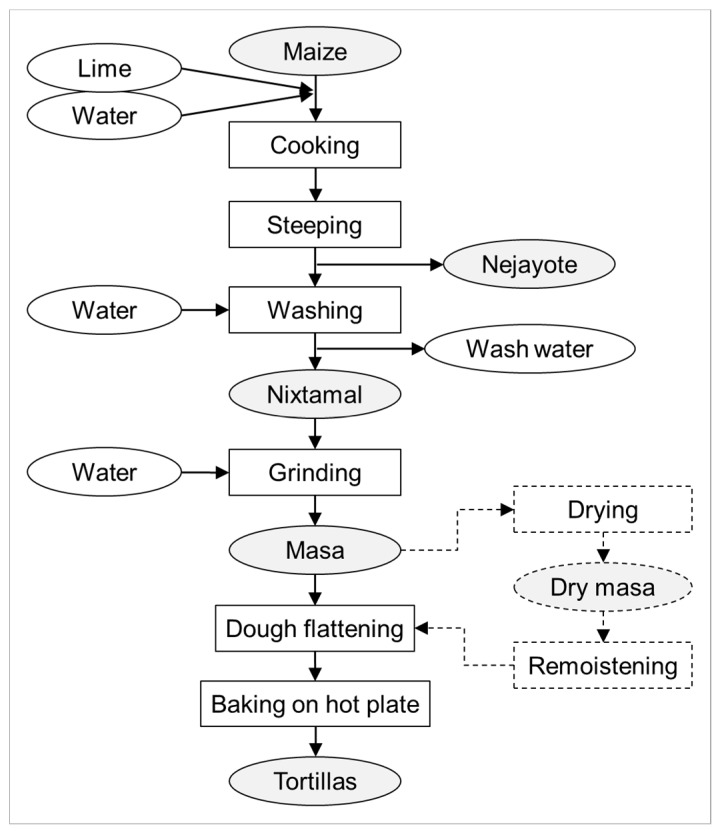
Scheme of typical steps and (by)products in traditional nixtamalization and tortilla production. The industrial production of tortillas often makes use of dry masa flour, which is made from dried nixtamal by fine grinding (not shown).

Nixtamalization describes an ancient food processing procedure developed and applied by indigenous Mesoamerican (e.g., Aztec and Mayan) civilizations [13], which is still used nowadays. It represents alkaline cooking of maize kernels. Traditionally, this is done using lime, which mainly consists of Ca(OH)_2_. Classic nixtamalization also makes use of wood ash. In traditional nixtamalization (Figure 1), maize kernels are cooked in lime water followed by steeping at room temperature, which typically takes place overnight. During nixtamalization, the elevated pH and high temperature facilitate the softening of the endosperm and the release of the pericarp. After steeping, the cooking and steeping liquid, the so-called nejayote, is removed. The alkaline-cooked kernels (nixtamal) are then washed with water to remove excessive lime, as well as (part of) the loosened pericarp. The aleurone layer, i.e., the outermost layer of the endosperm that is rich in protein and vitamin B1–3, stays attached to the starchy endosperm. The aleurone layer also contributes to a reduction of protein and starch losses during cooking, steeping, and washing [14]. After washing, the nixtamal is stone-ground upon the addition of water to form a maize dough called masa. Small portions of masa are formed into balls that are flattened into thin discs. The so-formed tortillas are baked on a hot plate or in an oven. By frying, tortillas and tortilla strips can be further processed into tostadas and tortilla chips (or similar products). Additionally, masa can also be used to prepare maize chips. With respect to storage, masa can be dried and later remoistened for further processing. Moreover, a dry masa flour can be produced from low-moisture nixtamal by fine grinding under dry conditions. In this process, due to the low moisture, no release of starch granules from the protein matrix occurs compared with fresh masa production, in which nixtamal is ground at a high moisture level. Dry masa flour is often used in commercial tortilla production.

Alkaline cooking of maize causes several physical, as well as (bio)chemical, changes [14,15]. Some of those are associated with the enhanced nutritional value of the grain and are of particular importance in diets mainly relying on maize. The improved bioaccessibility of calcium and niacin (vitamin B3) are likely the most important of these changes. Thanks to the latter, pellagra—a niacin-deficiency disease typically related to maize- and sorghum-based nutrition—is not common in tortilla-eating countries. Moreover, nixtamalization can improve protein digestibility and can positively affect the protein quality of maize by partially adjusting the leucine-to-isoleucine disproportion [16]. In tortillas, few amino acids were found to be reduced—including leucine, which can act as an antagonist of isoleucine. Nonetheless, maize protein is, in general, deficient in lysine and tryptophan but relatively rich in the sulfur-containing amino acids methionine and cystine. Nixtamalization furthermore enhances the content of resistant starch, which is accompanied by a lower glycemic index [17,18]—a factor that is nowadays of special interest. Furthermore, traditional tortilla production is capable of lowering mycotoxin contaminations, as outlined below, which is of particular significance for a healthy cereal-based diet. Lime-cooked maize and products thereof are, moreover, characterized by a unique flavor, which contributes to the increasing popularity of such foods on a global level [12,19].

## 3. Aflatoxins during Nixtamalization and Tortilla Production

### 3.1. Impact on Aflatoxin Concentrations by Traditional Nixtamalization

Because aflatoxins, particularly AFB1, are a severe health threat and are often found in maize produced in warmer climates, strategies for reducing aflatoxin contaminations are of special importance. Interestingly, traditional nixtamalization is in several studies described to have a high potential for lowering aflatoxin concentrations (for details, see Table 1). The nejayote, the main waste byproduct, which typically also contains a solid fraction that mainly consists of maize tip cap, pericarp, and germ, can in return show a certain accumulation of aflatoxins. 

The loss in aflatoxins and potential transfer to the lime water is also dependent on the type of aflatoxin [20,21]. In a study by Ulloa-Sosa et al. [21], AFB1 + AFB2 were less reduced (by around 40%–50% in nixtamal and masa) compared with AFG1 + AFG2 (by around 75%). The total aflatoxin concentration in nixtamal and masa was approximately reduced by 60%–65%. Remarkably, whereas most of the AFB1 + AFB2 were detected in the nejayote, no AFG1 or AFG2 could be detected in this matrix, pointing to a degradation or transformation into undetectable form(s) upon exposure to alkaline pH. Tortillas showed approximate reductions in total aflatoxins, AFB1 + AFB2, and AFG1 + AFG2 levels of 70%, 60%, and 80%, respectively. Additionally, de Arriola et al. [20] found AFG1 and AFG2 to be somewhat more reduced during nixtamalization than AFB1 and AFB2 (average reduction of AFB1, AFB2, AFG1, and AFG2 in masa approximately 93%, 90%, 98%, and 97%, respectively). Both studies were performed with fungal-inoculated kernels. Abbas et al. [22] analyzed the impact of the entire tortilla production process (covering nixtamalization using a 2% Ca(OH)_2_ solution) on natural aflatoxin contaminations. Here, the AFB1 content was on average reduced by 40%, and the AFB2 content was reduced by only 28%. AFG1 and AFG2 were not detected in unprocessed maize (or in tortillas). 

Enhancing the concentrations of Ca(OH)_2_ for cooking and steeping would not necessarily cause a more pronounced aflatoxin reduction in tortillas [20,23]. Lime concentrations of >2% are even described to produce tortillas with organoleptic characteristics unsuitable for human consumption [20]. De Arriola et al. [20] found average reductions of total aflatoxin concentrations in masa and tortillas of 94% and 95%, respectively, at 0.6% lime without significant difference compared with the use of 1.87% lime. The experiments were done on highly aflatoxin-contaminated maize obtained by fungal inoculation. Moreover, the cooking procedure (i.e., cooking in an open kettle versus pressure cooking in an autoclave) made no significant difference regarding the change in total aflatoxin concentration [20]. When comparing the impact of five different nixtamalization processes on aflatoxin concentrations (see Table 1), Price et al. [23] found cooking, prolonged steeping, and washing of nixtamal to facilitate aflatoxin reductions in nixtamal, masa, and tortillas. Here, aflatoxin levels were reduced by approximately 50%–70% in tortillas compared with the naturally contaminated unprocessed maize (initial aflatoxin level of around 140 µg/kg). When using AFB1-spiked kernels (100 µg/kg) in previous experiments, the authors mentioned that the toxin was almost not detected after tortilla production [23]. 

Based on their experimental data, Moreno-Pedraza et al. [24] proposed a (traditional) process for nixtamalization and tortilla production, which is supposed to completely reduce AFB1 contaminations. The key steps are as follows: (i) cooking kernels in 1% lime at 90 °C for 45 min; (ii) further steeping in the alkaline solution at 25 °C for 18 h; (iii) removal of the nejayote followed by only one washing step to remove the pericarp but to preserve the alkaline conditions (approximately pH 10) of the nixtamal; (iv) resting the masa, which has been obtained by grinding the washed nixtamal and still features the high pH value at room temperature for 40 min; and (v) flattening and cooking of the tortillas. The authors found that the nixtamalization step, the resting of the alkaline masa for ≥30 min, and/or the tortilla baking completely or almost completely eliminated AFB1 levels (either low natural contamination or a 115–125 µg/kg spike in the masa). In this study, the alkaline treatment lead to the formation of at least two unidentified degradation or transformation products: one of 301.25 Da (molecular formula: C_17_H_16_O_15_) and another of 325.33 Da (molecular formula: C_17_H_18_O_5_) [24]. 

To follow the fate of aflatoxin during traditional nixtamalization, radio-labeled AFB1 was used [25]. Natural AFB1 contamination (at low and high levels) was found to be lowered by 97%–100% after traditional nixtamalization using a chromatographic method. When radio-labeled AFB1 was spiked in unprocessed maize, the loss in radioactivity amounted to 84% in the masa. The remaining radioactivity was detected in the nejayote and the washing liquids (with decreasing levels in later washings). Whereas the aflatoxin retained in the masa appeared intact (detectable by an antibody-based method), aflatoxin in the liquid waste fractions was undetectable by means of chromatography [25].

### 3.2. Aflatoxin Reductions by Alternative Nixtamalization Processes

The abovementioned studies point to a high potential of traditional nixtamalization to lower aflatoxin concentrations. However, traditional nixtamalization is a time-consuming process characterized by a relatively high input of water and energy. Moreover, the nejayote is considered to be a highly problematic byproduct due to its excessive pH, its high content of organic and insoluble matter, and other factors [26], and it is usually disposed in landfills, not utilized [27]. Thus, to reduce water and energy inputs and the amount of wastewater, alternative processing procedures were considered. Such methods were not only tested regarding organoleptic and nutritional characteristics, but also in view of aflatoxin reductions as outlined below. 

One promising alternative method for masa and tortilla production might be the extrusion of maize meal upon the addition of lime. At a lime concentration of 0.2%, based on maize meal, organoleptic properties were found to be comparable to traditionally produced masa/tortillas (using a 0.33% lime solution, which represented 1% lime based on maize meal). Total protein and lysine contents were similar (or only slightly lowered), but the tryptophan loss was much lower in extruded masa compared with traditional masa [28]. A higher nutritional value, accompanied by an elevated weight gain and protein efficiency ratio in a rat feeding trial, was, in addition, shown for tortillas produced with maize meal extruded at 0.15%–0.25% lime (relative to maize meal mass) compared with traditional tortillas produced with masa prepared with 2% lime based on kernel mass (= 0.67% lime solution) [29]. Elias-Orozco et al. [30] evaluated the alkaline extrusion process regarding aflatoxin reductions in naturally contaminated maize. Astonishing, in the highly contaminated batch, they found a high level of aflatoxin M1 (AFM1) in the raw maize, which was only around 20% less than the AFB1 level. It had been previously described that *Aspergillus* spp. are capable of producing AFM1 (and aflatoxin M2); however, usually relatively low amounts of AFM1, compared with AFB1 or total aflatoxins, are found in maize or in culture media [31,32,33,34]. Furthermore, they detected AFB1-dihydrodiol in the raw maize, which is formed via enzymatic oxidation of AFB1 followed by non-enzymatic hydrolysis. Tortillas produced after extrusion of maize meal lacking any lime showed reductions in AFB1, AFB1-dihydrodiol, and AFM1 levels of approximately 46%, 54%, and 20%, respectively. When lime was added at 0.3% relative to maize meal mass, reductions increased to approximately 74%, 70%, and 52%, respectively. At 0.5% lime, they amounted to 83%–89%. Moreover, the authors tested extrusion upon the addition of 0.75%–3% H_2_O_2_, alone or in combination with 0.3% lime. Similar to lime, adding H_2_O_2_ to the extrusion process can increase aflatoxin reductions in tortillas. However, the combination of lime and H_2_O_2_ showed no or minor benefits compared with lime alone, except regarding AFM1 reduction. Here, reduction was enhanced from approximately 52% (0.3% lime, only) to 61%–73% (0.3% lime + 0.75%–3% H_2_O_2_ based on maize meal). To compare, traditional nixtamalization of the highly contaminated maize resulted in AFB1, AFB1-dihydrodiol, and AFM1 reductions of 92%–94%. Similar results on the effect of extrusion treatments and traditional nixtamalization were found for a maize batch contaminated with AFB1 at a lower level; other aflatoxins were here not detected [30] (see Table 1 for details).

Pérez-Flores et al. [26] tested the use of a microwave for the nixtamalization process. For that, maize grits (obtained from fungal-inoculated kernels) were cooked in a minimized amount of lime water (0.5% Ca(OH)_2_) in a microwave (for details, see Table 1). After steeping (3 h), no water removal or washing, which could have caused fractionation of mycotoxins, was indicated by the authors. The so-produced masa had 36%–82% lower aflatoxin concentrations than the maize kernels, with higher reduction at higher initial contamination levels. In tortillas, aflatoxin levels were lowered by 68%–84% of the initial amount. Tortillas produced by such microwave nixtamalization showed comparable physicochemical (moisture, pH, color) and technological properties (puffing, rollability, weight loss) as described for traditionally produced tortillas.

Minimization of water and energy input was also tested with a so-called ecological nixtamalization process [35]. Here, maize meal was mixed with a minimum amount of hot (92 °C) 0.375% lime solution for only 10 min. After steeping (2 h), the nixtamal was ground into masa without any water removal or washing steps. With such a process, AFB1 + AFB2 levels were lowered by 25%–40%, 13%–25%, and 61%–78% in the nixtamal, masa, and tortillas, respectively. Higher percentage reductions were, however, detected at lower initial contamination levels. Although applied to milled maize, the ecological nixtamalization was overall less effective in reducing aflatoxins compared with a traditional nixtamalization process applied to kernels of the same (fungal-inoculated) batch. The tested traditional nixtamalization covered a higher concentration of the lime water (1% lime), longer incubation times (70 min cooking, 12 h steeping), removal of the nejayote, and washing of the nixtamal (which typically also removes the loosened pericarp). Here, aflatoxin reductions amounted to 83%–92%, 87%–89%, and 90%–92% in nixtamal, masa, and tortillas [35].

Torres et al. [36] compared a traditional process (that included the cooking of kernels in lime water) with a commercial one. In the latter, whole maize kernels were mixed with lime and boiling water without further cooking (similar as described to the aforementioned ecological nixtamalization of maize meal [35]). In both processes tested by Torres et al. [36], the nejayote was removed after a 14 h steeping, and the nixtamal was washed twice. In the commercial process, the pH levels of the nixtamal, masa, and tortillas were lower compared with those in the traditional one (5.8–5.9 compared to 6.7–6.8). Moisture content was also slightly lower. Further, the loss of solids was reduced (4.1% in the commercial versus 6.8% in the traditional process). These factors likely contributed to the lower efficiency of aflatoxin reduction: The commercial processing reduced the level of total aflatoxins in tortillas by 30%, whereas the traditional tortilla production was more efficient (52% aflatoxin loss). Maize chips and tortilla chips showed aflatoxin reductions of 71% when using traditionally produced masa and 79%–85% upon use of masa produced with the tested commercial process. However, maize at different initial aflatoxin concentrations was applied to the two processes, which might also have affected the aflatoxin reduction efficiency. In this study, samples were acidified upon extraction (before filtration of suspended samples) to cause a reconversion of potential transiently transformed aflatoxins [36] (see below).

### 3.3. Potential Reconversion of Modified Aflatoxins

In general, besides the leaching of aflatoxins into liquid fractions, alkaline conditions can cause the opening of the lactone ring of aflatoxins (including AFB1), resulting in a loss of fluorescence of the molecules and thus a loss of fluorescence-based detection. Further, a strongly reduced toxicity and mutagenicity after cleavage of the lactone ring was described [37]. In nixtamalized maize (products), the lowered aflatoxin concentrations were found to be accompanied by lower mutagenicity and oxidative stress in vitro [23,38]. Vázquez-Durán et al. [38] showed that for extracts of raw maize, a more pronounced lipid peroxidation in kidney Vero cells occurred than for extracts of tortillas, which were produced from the raw maize by a microwave nixtamalization process (as described by Pérez-Flores et al. [26]) and had a 84% lower aflatoxin level. Further, no mutagenic toxicity was detected in the tortillas in the Ames test, but it was present in the unprocessed maize. Similarly, Price and Jorgensen [23] observed a reduced mutagenic potential for masa and tortilla samples compared with raw maize when testing different nixtamalization processes (although the number of revertants in the Ames test did not always correlate with the detected aflatoxin level regarding the extent of reductions).

The modification of aflatoxins during alkaline treatment is not necessarily permanent, however, and might be reversed upon exposure to acidic conditions, as present in monogastric digestive systems. Price and Jorgensen addressed this issue by acidifying the suspended samples in the course of aflatoxin extraction (original pH around 11; acidified: 5–6), mimicking acidification in the human stomach. In fact, in doing so, part of the undetectable modified aflatoxin(s) was reconverted into fluorescent form(s). After acidification, the total aflatoxin reduction in the tortillas amounted to only 20%–46%, instead of 48%–73% when lacking such a step. For masa, the reduction in fluorescent aflatoxin(s) was approximately 14%–56% without and 4%–29% with acidification. Additionally, Méndez-Albores et al. [39] showed that the reduction in aflatoxin concentrations by nixtamalization is partly reversible. After acidification, aflatoxins became, to some extent (approximately 5% of the concentration in raw maize), detectable in the dried nejayote, which originally had a pH of 12. When the extracts of the samples were acidified (initial pH of samples: 8.2–8.3), aflatoxin concentrations were around 57% and 34% higher compared with those in the alkaline extracts of masa and tortilla, respectively. However, compared with the raw maize, the aflatoxin levels were still very low with reductions of 78% in masa and 91% in tortillas (for the alkaline extracts, reductions amounted to 86% and 93%, respectively). Pérez-Flores et al. [26] found that only very low amounts of aflatoxins in the extracts of masa and tortillas were recovered by an acidification step. Here, the pH of masa and tortillas was again around 8.2 prior acidification (and adjusted to 3). Different from Price and Jorgensen [23], in the two latter studies [26,39], not the suspended samples, but the sample extracts were acidified. However, when treated with weak bases and during ammoniation, AFB1 was found to interact with matrix macromolecules, including non-protein fractions [40,41]. Hence, it is tempting to speculate that the extraction efficiency of the modified aflatoxin(s) is dependent on the pH of the matrix and that it is higher under acidic conditions. If this is true, matrix-associated aflatoxins potentially present in tortillas could be also released in the stomach.

In the study by Price and Jorgensen [23], acidification of the samples was found to be further capable of restoring mutagenicity in the Ames test. The mutagenic effects for the tortilla samples were even somewhat higher than for raw maize, which, however, contradicted the reduced aflatoxin concentrations that were observed in the acidified tortilla samples [23]. This might indicate the formation of additional mutagenic form(s) during tortilla production, which could also explain the rather low correlation between mutagenicity and aflatoxin concentration determined by the authors. In general, the efficiency and persistency of aflatoxin transformation/detoxification by elevated pH is dependent on several factors. Positive effects of temperature, time, and kernel moisture on AFB1 reduction under alkaline conditions were shown for ammoniation at atmospheric pressure [42] and under elevated pressure [43]. Differences in aflatoxin reductions depending on the processing procedures were also found for nixtamalization and tortilla production (Table 1). Further, initial contamination levels and type of contamination (contamination in the field, post-harvest contamination, spiking with pure standard) might affect mycotoxin reduction efficiencies. Such factors should be considered when assessing the aflatoxin loss in view of toxicity in alkaline-processed maize and products thereof, such as tortillas.

## 4. Fumonisins during Nixtamalization and Tortilla Production

### 4.1. General Impact on Fumonisin Concentrations and Fumonisin Hydrolyzation

Fumonisins are very water-soluble mycotoxins, which can thus leach into the liquid fraction during cooking and steeping procedures. Furthermore, an alkaline treatment can result in a hydrolysis of the *O*-acyl bonds of fumonisins, leading to the formation of hydrolyzed fumonisins. Sydenham et al. [44] found, upon steeping of maize kernels and maize meal in 0.1 M Ca(OH)_2_ (at room temperature, under continuous stirring), a reduction in FB1 concentrations and an accumulation of fully hydrolyzed FB1 (HFB1; also referred to as aminopentol). For maize meal naturally contaminated with FB1, almost all of the mycotoxin was lost. Here, around 78%–89% of the FB1 was converted into HFB1, with 68%–72% being transferred into the steeping liquid and 11%–17% remaining in the alkali-treated maize meal. The latter contained only up to 9% of the FB1 level of untreated maize meal. In total, around 11%–25% of the FB1 was retained as FB1 or HFB1 in the maize meal. When treating whole kernels (also naturally contaminated) in the same manner, the reduction in FB1 concentration amounted to 76%–99%. After treatment, kernels were manually sorted by the extent of pericarp loss. Kernels with fully removed pericarp showed almost no FB1 left, and only approximately 4% of the FB1 was detected to be present as HFB1. In kernels with partly removed pericarp, approximately 7% of the initial FB1 was present as HFB1, and 24% remained in the parent form [44]. Accordingly, the removal of the nejayote and of maize pericarp would contribute to fumonisin reduction in nixtamalized maize.

Pilot-scale processing of naturally contaminated maize simulating commercial nixtamalization and tortilla (chips) production showed significant reductions in concentrations of FB1 and FB2 [45,46]. Although having a similar pattern, the extent of fumonisin reduction varied in both studies among individual runs, independent of initial concentration. Voss et al. [45] used different maize batches for five runs. Dombrink-Kurtzman et al. [46] examined the same maize batch in two runs but found nonetheless strong variations, particularly for FB2 (for details, see Table 2). FB1 reduction in nixtamal was in both studies accompanied by an accumulation of HFB1 in the steeping and washing liquids. Voss et al. [45] detected, besides HFB1, some partially hydrolyzed FB1 (PHFB1) in the raw maize. However, this compound did not accumulate in the nejayote but, if present, decreased over time. The decrease in FB1 and PHFB1 was accompanied by an increase in the fully hydrolyzed form. In general, hydrolyzation particularly takes place in the nejayote, which typically has a pH of ≥11. Palencia et al. [47] found the molar ratio of HFB1 to FB1 to be 21 in the nejayote but around 1 in wash water, masa, and tortillas. The overall transfer of fumonisins to the nejayote amounted in the study of Voss et al. [45] to approximately 45% of the total initial amount (on a molar basis) of FB1, PHFB1, and HFB1. Additional amounts were detected in the washing water. Dombrink-Kurtzman et al. [46] described the liquid fractions to contain on average of 76% of the initial FB1: 72.5% of FB1 was converted into HFB1 and 3.5% remained as FB1. The study also indicated the potential for further lowering of FB1 and FB2 levels during masa/tortilla production, in case they were somewhat less reduced in the nixtamal. FB1 and FB2 reductions in tortillas amounted in both runs to 88%–92% and 71%–91% compared with the levels in unprocessed maize, respectively. However, in one of the runs, the nixtamal showed reductions of around 75% for FB1 and only 20%–30% for FB2 [46] (Table 2). 

The potential impact of tortilla baking on fumonisin reductions also became obvious in a study that tested a microwave nixtamalization process using maize grits. Here, total fumonisin levels were not significantly lowered in masa. This was in accordance with the tested processing procedure, because no removal of nejayote or washing of nixtamal was indicated by the authors. Different from masa production, the baking of tortilla lowered fumonisins by approximately one half. The reduction during the heat treatment was likely facilitated by the high pH of masa and tortillas (i.e., around 8.1–8.3). The tested physicochemical and technological characteristics were similar to those described for traditionally produced tortillas [48].

The fate of fumonisins was also investigated during commercial processing into tortilla (chips) [49,50]. Scudamore et al. [49] analyzed industrial tortilla chip production in United Kingdom (UK) plants, involving mixing of a maize flour dough followed by sheeting, cutting, baking, and frying. Alkaline conditions were, however, not indicated. However, because nixtamalization contributes to the typical flavor of tortillas and tortilla chips, we assume that dry masa flour or a similar ingredient was involved in the commercial production process. When analyzing 11 runs (that comprised two different compositions of maize flour mixtures), FB1 + FB2 were lowered by 32%–78% on the product ‘as is’ basis (average: 59%). Because the moisture content of the chips is usually more or less comparable to that of dry maize ingredients, a similar fumonisin reduction would apply when related to dry weight. Commercial tortilla production in Texas was studied by De La Campa et al. [50]. Here, the reduction in FB1 levels was high overall and ranged from 80% to 100% in masa and from 83% to 100% in tortillas. Production conditions strongly differed between the four processing plants, regarding, for example, lime concentration and cooking time. 

In experimental studies, De La Campa et al. [50] further investigated the impact of these factors at different initial FB1 levels using fungal-inoculated maize. In doing so, they found a positive impact of lime concentration (when testing lime solutions of around 0.25%–1.6%) on FB1 reduction. This effect was independent of the initial FB1 concentration, which also had a significant effect on FB1 half-life. Regarding boiling time (15 versus 60 min), the authors mentioned that this factor had no apparent effect, but data were not shown [50]. Additionally, De Girolamo et al. [51] described the low effect of cooking time on the hydrolyzation of fumonisins, when comparing cooking times of 15, 30, and 60 min. In this study, which tested lime solutions with concentrations of around 0.33% and 1.67%, nixtamalization lowered mean FB1 + FB2 levels in masa by 26%–48%. Interestingly, the same process lacking lime resulted in a somehow stronger FB1 + FB2 reduction. Here, PHFB (PHFB1 + PHFB2) levels in masa were also lowered, but the loss was not accompanied by the formation of HFBs (HFB1 + HFB2). Reductions are likely solely caused by the leaching of fumonisins into the liquid fractions. By contrast, the use of lime provoked the formation of (partially) hydrolyzed forms of FB1 and FB2. Hydrolyzation was again more pronounced at higher lime concentration [51]. 

### 4.2. Potential Further Transformations of Fumonisins

On the one hand, De Girolamo et al. [51] discovered that alkaline cooking can somehow facilitate the release of bound fumonisins. In their study, the total mass of FB1 + FB2, PHFBs, and HFBs recovered after nixtamalization (also including the liquid waste fractions) exceeded the initial mass by around 50%–80%. In the course of nixtamalization, (part of) the released matrix-associated fumonisins were suggested to be hydrolyzed. Although the water-cooked maize showed a higher reduction in (free) FB1 + FB2 than the alkali-cooked maize, matrix-associated fumonisins would still be present. Moreover, bound fumonisins in food (and feed) products can, in general, increase health concerns, because free toxins might be released during digestion. Promoting the release from the matrix followed by hydrolyzation of fumonisins could contribute to a detoxification by nixtamalization. Different from FB1 and FB2, no (liver) cancer-promoting activity or weight loss was found for HFB1 and HFB2 in rats [52]. In contrast, in vitro tests showed a higher toxicity on primary rat hepatocytes in this study. Hence, it was concluded that the hydrolyzed fumonisins are not adsorbed from the gut [52]. A lower or lacking hepatic, intestinal, and neural toxicity of the hydrolyzed form compared with the parent compound was also shown in pigs and mice [53,54], although an impact on sphingolipid metabolisms in vivo was demonstrated at a high dose of HFB1 [54]. Inhibition of ceramide synthase and disruption of sphingolipid metabolism is the critical biochemical effect underlying fumonisin cytotoxicity.

On the other hand, hydrolyzation might also favor interaction with other compounds, including matrix macromolecules. Interestingly, Park et al. [55] were able to detect matrix-associated fumonisins in some retail tortilla chip samples. However, when analyzing retail samples, it cannot be excluded that the forms were already present in the raw material. To address this question, Burns [56] investigated a nixtamalization process by applying the detection method developed by Park et al. [55]. In doing so, a significant increase in protein-bound and other matrix-associated FB1 during nixtamalization was demonstrated. When maize kernels were processed in the same manner, but lacking lime, no significant change was observed in the concentration of total matrix-associated FB1 [56]. A reduction in recoverable (H)FB1 was described for experimentally produced and extruded masa flour [57]. However, here, the underlying mechanisms (degradation, binding, or modification to undetected free forms) remained unknown. 

*N*-(carboxymethyl)-FB1 was previously shown to be formed under alkaline conditions at elevated temperatures by using pure FB1 incubated overnight with D-glucose [58] and also when heating HFB1 with D-glucose [59]. Interaction with glucose during extrusion cooking of maize resulted in a strong reduction in fumonisin-induced toxicity in rats [60]. However, when analyzing nixtamalization and tortilla chip production mimicking commercial processing, no indications were given for a (relevant) formation and accumulation of fumonisin–sugar adducts, namely *N*-(carboxymethyl)-FB1 and *N*-(1-deoxy-D-fructos-1-yl)-FB1 [45]. By contrast, Park et al. [61] could detect *N*-fatty acyl fumonisins in a tortilla chip sample (in 1 out of 38 retail samples), indicating a potential formation of those modified fumonisins in alkali-treated and fried maize products. In vitro studies implicate a high toxicity of several *N*-fatty acyl fumonisins. In addition, such modified forms can be more rapidly taken up and accumulated in human/animal cells than FB1 (for an overview, see [4]). However, further studies are required to obtain more information on the toxicity of *N*-acetylated fumonisins and their occurrence in foods. The same is true for the interaction of fumonisins with other molecules that potentially takes place during nixtamalization and the possible contribution to fumonisin-related toxicity. 

Using bioassays and feeding trials, several studies indicate a reduced toxicity of FB1-contaminated maize raw material after being processed by nixtamalization [47,62,63,64]. To analyze (potential) kidney damage, in addition to histological analysis, sphinganine can be used as a biomarker for fumonisin-induced ceramide synthase inhibition. Palencia et al. [47] detected reduced accumulation of sphinganine in cell lines treated with extracts of tortillas compared with those treated with extracts of raw maize. This was in conjunction with lowered FB1 levels. Here, the sum of FB1 and HFB1 in tortillas (on molar basis) was half of the initial FB1 level detected in the raw maize. The toxic potential was found to be lowered by 60% for extracts of tortillas compared with extracts of unprocessed maize [47]. Similarly, in feeding trials on rats, kidney sphinganine and sphingosine concentrations were not increased or less increased in rats fed a diet containing nixtamalized maize (meal) compared with those fed non-nixtamalized maize (meal) [62,63]. In both studies, nixtamalization was performed with a 1.2% lime solution. Voss et al. [63] used raw maize with three different FB1 contamination levels. Rats that ate a diet containing nixtamalized maize showed no or only week symptoms of nephropathy. This was much different from when the diet contained uncooked maize. Burns et al. [62] additionally included a mock-nixtamalization control (i.e., cooking of maize meal without lime). Similar to the findings of De Girolamo et al. [51], this procedure also lowered the FB1 level, but much less hydrolyzation took place compared with cooking with lime (for details, see Table 2). Both nixtamalization and mock-nixtamalization strongly reduced kidney damage, as well as renal toxicity (evaluated by number of apoptotic tubule cells), compared with uncooked maize meal [62]. Due to the clearly reduced toxic effects caused by nixtamalized maize (products), a significant formation of matrix-associated FB1 or unknown free fumonisin forms that contributed to toxicity was not indicated in these two studies. This differed from a former rat feeding trial performed by Hendrich et al. [64] using highly FB1-contaminated maize (obtained by fungal inoculation). Here, although nixtamalization was able to lower toxicity in some cases, a more pronounced effect would be expected in view of the high loss in FB1 (approximately 98%–100%). When considering the molecular weights, the formed HFB1 amounted to approximately 60%–72% of the initial FB1. In this study, it became further obvious that the nutritional status was capable of impacting toxicological effects caused by fumonisins present in the non-nixtamalized and nixtamalized maize. Hence, more research is needed regarding the potential formation and occurrence of so far undetected and/or unknown toxic fumonisin form(s) in alkali-cooked maize.

## 5. Other Mycotoxins during Nixtamalization and Tortilla Production

Little data were found on mycotoxins other than aflatoxins and fumonisins during nixtamalization and tortilla production (Table 3). Abbas et al. [65] experimentally produced tortillas (including traditional nixtamalization with 2% Ca(OH)_2_) using two batches of maize naturally contaminated with the *Fusarium* toxins ZEN and DON, as well as maize spiked with the purified toxins (by injection into the embryos). Here, no difference in the percentage reduction of mycotoxin was obvious depending on the type of contamination. For ZEN, the initial levels that were present as *trans*-ZEN were lowered by 59%–100%. For the two maize samples with the highest concentrations (one spiked and one naturally contaminated one), some ZEN (<0.4% of the total amount) could be detected in the nejayote. Further, some isomerization from *trans*-ZEN to *cis*-ZEN took place for these maize samples. However, most of the ZEN was degraded into undetectable form(s), and it was supposed that the alkaline treatment attacked the lactone ring of ZEN. Whether this transformation would be stable under acidic conditions was not addressed [65]. For DON, reductions amounted to 72%–82%. The naturally contaminated maize batches contained, in addition, the acetylated form 15-acetyl-DON, which was completely destroyed in tortillas. Neither DON nor 15-acetyl-DON could be detected in the nejayote. 

The potential to lower ZEN and DON by alkaline steeping of maize was also shown when using 0.1 M sodium carbonate. Here, steeping of raw maize kernels at 22 °C for 24 h lowered ZEN and DON by around 45% and 70%, respectively. An extended steeping over 72 h reduced the concentrations by 88% and 95%, respectively [66]. The baking and frying steps in commercial tortilla chip production were analyzed by Scudamore et al. [49] regarding mycotoxin changes on a product ‘as is’ basis. In the (probably alkaline) maize flour mixture(s) used to prepare the dough for tortillas, ZEN was present at low levels only, and the change during processing was very variable. However, if the initial ZEN level was higher than 13 µg/kg, the reduction amounted to 35%–64%. If the initial level was below 9 µg/kg, the detected change ranged from a 7% reduction to a 116% increase. This was probably caused by difficulties in representative sampling of industrial processes. DON levels in the tortilla chips were on average lowered by 32%, with the highest reductions at the highest initial levels. The sensitivity of DON towards food production processes that involve alkaline additives was also observed in the production of bakery wares and during the cooking of noodles (for an overview, see [67]). 

The reduction of the emerging mycotoxin MON during tortilla production was studied by Pineda-Valdes et al. [68]. In pilot-scale experiments, MON was reduced by 97% after cooking of maize kernels in a 0.25% lime solution. After steeping or further processing, MON could not be detected anymore. When determining laboratory-scale processing using fungal-inoculated maize with an around 10-fold higher initial concentration, MON was lowered by 54% during cooking. After steeping and washing, MON reduction accounted for 64% and 69%, respectively. In masa and tortillas, the loss was around 70%. MON was not detected in any of the liquid fractions [68], although it is characterized by low molecular size and high water solubility [8]. Thus, MON might have been either modified into undetected form(s) during the 20 min alkaline cooking step or was degraded due to the action of high temperature and/or high pH. In a former study, Pineda-Valdes and Bullerman [69] demonstrated an affection of MON at elevated pH and temperature. Heating to 100 °C in an aqueous environment with pH 10 for 60 min lowered MON by around one half. However, after 20 min of cooking, the MON loss amounted to less than 20%. Therefore, nixtamalization of MON-contaminated maize showed a relatively high efficiency in reducing the concentration of this emerging mycotoxin, probably by a pH of >10 of the lime water.

## 6. Conclusions and Outlook

Nixtamalization and tortilla baking can affect mycotoxins in different ways, including physical and chemical action: (1) Water-soluble mycotoxins can leach into the liquid fractions during cooking, steeping, and washing. (2) Mycotoxins present in the pericarp, tip cap, and germ are removed when these tissues are (partly) separated by thorough washing of the nixtamal. (3) Action of high pH and elevated temperature during cooking and baking can result in degradation, modification, and/or binding or release of mycotoxins.

**Figure 2:**
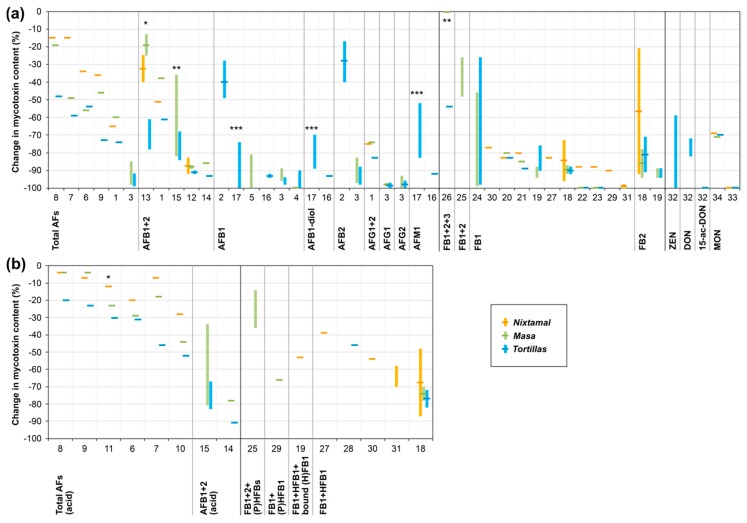
Graphical overview of mycotoxin changes during nixtamalization and tortilla production. (**a**) Free parent mycotoxins. (**b**) Sum of free parent form(s) and detected modified/ matrix-associated form(s). Columns indicate (approximate) ranges; lines represent (approximate) mean values of changes in mycotoxin concentrations from raw maize material to tortillas or intermediate products. The numbers on the x-axis refer to studies described in the literature, which are listed in Table 1, Table 2 and Table 3. More than one number can refer to the same reference if different process conditions or technologies were compared. For details on the studies (including references, processing parameters, and mycotoxin content in raw maize material), see Table 1, Table 2 and Table 3. *: Only mixing of kernels with hot lime water, without further cooking. **: Microwave cooking. ***: Extrusion cooking. Total AFs: aflatoxins B1 + B2 + G1 + G2; Total AFs (acid): aflatoxins B1 + B2 + G1 + G2 detected in acidified samples/extracts; AFB1/2: aflatoxin B1/2; AFB1 + 2 (acid): aflatoxin B1 + B2 detected in acidified samples/extracts; AFB1-diol: aflatoxin B1 dihydrodiol; AFG1/2: aflatoxin G1/2; AFM1: aflatoxin M1; FB1/2/3: fumonisin B1/2/3; (P)HFBs: partly + fully hydrolyzed fumonisins B1 + B2; (P)HFB1: partly + fully hydrolyzed fumonisin B1; ZEN: zearalenone; DON: deoxynivalenol; 15-ac-DON: 15-acetyl-deoxynivalenol; MON: moniliformin.

For traditional nixtamalization, a high potential to lower free parent forms of mycotoxins is described (see also Figure 2). Aflatoxin concentrations of raw maize were found to be lowered by around 15%–85% and 20%–100% in the nixtamal and masa, respectively. Tortillas mostly showed aflatoxin reductions of 50%–100%. For FB1, the reduction mainly amounted to around 75%–100% in nixtamal, masa, and tortillas. For ZEN, DON, and MON, reductions of around 60%–100%, 70%–80%, and 70%–100% are described. However, only very limited data is available regarding maize mycotoxins other than aflatoxins and fumonisins. More data on such toxins would help to evaluate the benefits of alkali-processed maize in more detail. 

Besides reduction in the free parent forms, modification of mycotoxins can occur, and interaction with matrix compounds can be altered. To analyze such processes during nixtamalization, intense efforts have already been undertaken to establish and optimize appropriate detection methods. Although it must be noted that, when analyzing food matrices, which can harbor several challenges, analytical recovery must in general be taken into account, and data should be corrected accordingly (which was often not done or at least not mentioned for the data presented in the current review). Careful conclusions on the reduction factors of mycotoxins must certainly also take a reasonable contribution of variability and uncertainty into account. Furthermore, the stability of the present forms and their bioavailability need to be considered when analyzing toxicological impacts. In addition, precise knowledge on the critical parameters in nixtamalization and tortilla production is important to optimize production procedures to furthermore reduce potential health risks to the consumers, e.g., by reconversion of aflatoxins after consumption.

In general, further research is needed to evaluate possible modifications and matrix–mycotoxin interactions during nixtamalization, as well as the occurrence and potential toxicity of the formed structures in the final food items. In doing so, a possible reconversion and/or release of parent forms in the gastrointestinal tract, as well as by activity of the gut microflora, need to be considered. Reliable analytical data would be the basis for precise understanding of the processes and the factors in mycotoxin reduction. Moreover, knowledge on the fate of mycotoxins and their toxicity is required to evaluate possible utilization strategies for the nejayote.

## Figures and Tables

**Table 1:** Effect of alkaline cooking (nixtamalization) of maize kernels and of entire tortilla (chips) production on aflatoxin contents.

Study No. in Figure 2	Nixtamalization					Tortilla Baking on Hot Plate	Afla- toxin(s)	Initial Level in Raw Maize (µg/kg) ^a^	Corrected Change (%) ^b^	Comment(s)	Reference
	Alkaline Solution	Additions on Orig. Maize Mass Basis	Cooking	Steeping	Washing of Nixtamal						
1	ca. 7.5% lime solution	167% water, ca. 12.5% lime	60 min	Only cool down after cooking	Not indicated	Performed (further details not provided)	Total AFs	Not provided (inoculated)	Nixtamal: ca. −65 ^c^ Nejayote: accum. Masa: ~−60 ^c^ Tortillas: ~−74 ^c^	Overall: Relative quantification only and very approximate data	[21]
							AFB1 + AFB2	Not provided (inoculated)	Nixtamal: ~−51 ^c^ Nejayote: accum. Masa: ~−38 ^c^ Tortillas: ~−61 ^c^	Most of the lost AFB was found in the nejayote	
							AFG1 + AFG2	Not provided (inoculated)	Nixtamal: ~−75 ^c^ Nejayote: n.d. Masa: ~−74 ^c^ Tortillas: ~−83 ^c^	AFG1 + AFG2 were largely decomposed	
2	2% Ca(OH)_2_ solution	Not specified	5 min (incl. stirring)	12 h	Thorough rinse with distilled water	110–120 °C, 7–8 min on each side	AFB1	417–476	Tortillas: −28 to −49 (mean: −40)	-	[22]
							AFB2	54–59	Tortillas: −17 to −40 (mean: −28)	-	
3	0.6%–1.87% lime solution	1480% water, 1–3% lime	95 °C, 40 min or 121 °C, 0.34 bar, 30 min	O/N	Several washes with tap water	180–250 °C (internal temp.: 94 °C), 1.5 min	Total AFs	5245–60,478 (inoculated)	Masa: ~−85 to −98 Tortillas: ~−92 to −99	No significant differences between different treatments	[20]
							AFB1	3265–37,696 (inoculated)	Masa: ~−89 to −96 Tortillas: ~−94 to −98		
							AFB2	512–9513 (inoculated)	Masa: ~−83 to −97 Tortillas: ~−88 to −98		
							AFG1	1236–11,131 (inoculated)	Masa: ~−97 to −99 Tortillas: ~−97 to −100		
							AFG2	226–1639 (inoculated)	Masa: ~−93 to −100 Tortillas: ~−96 to −100		
4	0.33% lime solution	300% water, 1% lime	90 °C, 45 min	24 °C, 18 h	1 rinse with tap water	-	AFB1	0.68	Masa: ca. −100 (<LOD) ^c^	-	[24]
						150 °C, 5 min on each side	AFB1	125 (spiked to masa)	Tortillas: ca. −90 to −100 ^c^	Range is due to different detection methods	
5	Lime solution	1% lime (water: not specified)	94 °C, 50 min	17 h	2–3 washes	-	AFB1	37|251	Masa: −100|−97 ^d^	-	[25]
							^3^H-AFB1	~200 (spiked)	Masa: −81 to −84 ^d^ Nejayote: accum. Washing water: accum.	Range is due to different detection methods	
6	0.33% Ca(OH)_2_ solution	300% water, 1% Ca(OH)_2_	20 min	15 h	Thorough rinse with water	1 min on each side	AF	135	Nixtamal: ~−34/~−20 * Masa: ~−56/~−29 * Tortillas: ~−54/~−31 *	-	[23]
7	As described above	As described above	As described above	As described above	As described above	As described above	AF	142	Nixtamal: ~−15/~−7 * Masa: ~−49/~−18 * Tortillas: ~−59/~−46 *	Steeping was performed before cooking!	
-	As described above	As described above	-	As described above	As described above	As described above	AF	145	Nixtamal: ~−17/~−6 * Masa: ~−14/~−4 * Tortillas: ~−54/~−42 *	-	
8	7.8% Ca(OH)_2_ solution	160% water, 12.5% Ca(OH)_2_	60 min	Left to cool down for 1 h	As described above	As described above	AF	142	Nixtamal: ~−15/~−4 * Masa: ~−19/~−4 * Tortillas: ~−48/~−20 *	-	
9	0.25% Ca(OH)_2_ solution	300% water, 0.75% Ca(OH)_2_	75 min	24 h	-	As described above	AF	142	Nixtamal: ~−36/~−7 * Masa: ~−46/~−4 * Tortillas: ~−73/~−23 *	-	
10	0.33% lime solution	300% water, 1% lime	98 °C, 40 min	14 h	2 washes with 300% water (based on orig. maize mass)	Baking in a three-tiered, gas-fired oven for 39 s. Average temp. at the three levels: 177 °C, 233 °C, 453 °C	Total AFs	ca. 110	Nixtamal: ~−28 * Nejayote: n.d. * Masa: ~−44 * Tortillas: −52 * Maize chips: −79 * Tortilla chips: −85 *	Both processes: Corn chips and tortilla chips were prepared by frying masa and tortilla strips in oil at 190 °C for 2 and 3 min, respectively	[36]
11	As described above	As described above	No cooking, but manual mixing of maize and lime with boiling water	As described above	As described above	As described above	Total AFs	ca. 43	Nixtamal: ~−12 * Nejayote: accum. * Masa: ~−23 * Tortillas: −30 * Maize chips: −71 * Tortilla chips: −71 *		
12	1% lime solution	200% distilled water, 2% lime	85 °C, 70 min	22 °C, 12 h	1 wash with 200% tap water (based on orig. maize mass)	270 °C, in total 50–54 s on each side	AFB1 + AFB2	29|93 (inoculated)	Nixtamal: −92|−83 ^c^ Masa: −89|−87 ^c^ Tortillas: −92|−90 ^c^	―	[35]
13	0.375% lime solution	80% distilled water, 0.3% lime	No cooking, but manual mixing of maize and hot lime water (92 °C) for 10 min	22 °C, 2 h; no removal of water indicated	-	As described above	AFB1 + AFB2	29|93 (inoculated)	Nixtamal: −40|−25 ^c^ Masa: −25|−13 ^c^ Tortillas: −78|−61 ^c^	Use of maize meal (particle size: 800 µm)	
14	1% lime solution	300% distilled water, 3% lime	85 °C, 35 min	22 °C, 14 h	1 wash 200% with tap water (based on orig. maize mass)	270 °C, in total 50–54 s on each side	AFB1 + AFB2	678/680 ** (inoculated)	Nejayote: n.d./slight accum. ** Masa: ~−86/~−78 ** Tortillas: −93/~−91 **	Most aflatoxins appear to be degraded	[39]
15	0.5% Ca(OH)_2_ solution	100% water, 0.5% Ca(OH)_2_	Microwave (1650 W, 2450 Hz), 5.5 min	22 °C, 3 h; no water removal	-	270 °C, in total 54–55 s on each side	AFB1 + AFB2	22–141 (inoculated)	Masa: −36 to −82/~−34 to −81 ** Tortillas: −68 to −84/~−67 to −83 **	Use of maize grits. Higher reduction at higher initial concentration	[26]
16	1% lime solution	300% water, 3% lime	90–96 °C, 30 min	O/N	Several rinses with tap water	290 °C, in total 40–80 s on each side	AFB1	495|29	Tortillas: −94|~−92	-	[30]
							AFB1-diol	30|-	Tortillas: ~−93|-		
							AFM1	402|-	Tortillas: −92|-		
-	Water, only	75% water	Extruder (low shear, single- screw, 35 rpm screw speed, 87 °C barrel temp.)	-	-	As described above	AFB1	495|29	Tortillas: ~−46|~−68	All extrusion treatments: Use of maize meal (particle size: 800 µm)	
							AFB1-diol	30|-	Tortillas: −54|-		
							AFM1	402|-	Tortillas: −20|-		
17	0.4%–0.67% lime solution	75% water, 0.3%–0.5% lime	Extruder (for details see above)	-	-	As described above	AFB1	495|29	Tortillas: −74 to −85|~−100	Higher reduction at higher lime concentration	
							AFB1-diol	30|-	Tortillas: ~−70 to −89|-		
							AFM1	402|-	Tortillas: ~−52 to −83|-		
-	0.4% lime, 1%–4% H_2_O_2_ solution	75% water, 0.3% lime, 0.75%–3% H_2_O_2_	Extruder (for details see above)	-	-	As described above	AFB1	495|29	Tortillas: ~−67 to −78|~−100	Higher reduction at higher H_2_O_2_ concentration. At 0.3% lime + 3% H_2_O_2_: affection of taste	
							AFB1-diol	30|-	Tortillas: ~−68 to −84|-		
							AFM1	402|-	Tortillas: ~−69 to −81|-		

^a^: If not mentioned otherwise, maize was naturally contaminated. ^b^: Unless indicated otherwise, the change in the mycotoxin concentration is corrected for change in moisture content. Negative values: reduction; positive values: increase. ^c^: Here, it is not clear if the change in the mycotoxin concentration is corrected for change in moisture. ^d^: Here, change in the mycotoxin concentration is supposed to be corrected for change in moisture. *: Here, aflatoxin detection involved acidification of the suspended sample. Values before the forward slash (if present) are derived from analyzing samples without acidification. **: Here, aflatoxin detection involved acidification of the extracts. Values before the forward slash are derived from analyzing extracts before acidification. ~: Approximate values that were calculated for this overview by using the data provided in the cited literature. |: Here, individual data of two batches are given and separated by this symbol. AFB1: aflatoxin B1; AFB1-diol: aflatoxin B1 dihydrodiol; AF: aflatoxin(s) not further specified in the cited study but likely total aflatoxins B1 + B2 + G1 + G2; total AFs: aflatoxins B1 + B2 + G1 + G2. accum.: accumulation; LOD: limit of detection; n.d.: not detected; O/N: overnight; orig.: original.

**Table 2:** Effect of alkaline cooking (nixtamalization) of maize kernels and of entire tortilla (chips) production on fumonisin contents.

Study No. in Figure 2	Nixtamalization					Tortilla Baking on Hot Plate	Fumo- nisin(s)	Initial level in Raw Maize (µg/kg) ^a^	Corrected Change (%) ^b^	Comment(s)	Reference
	Alkaline Solution	Additions on Orig. Maize Mass Basis	Cooking	Steeping	Washing of Nixtamal						
18	~0.37% lime solution	~1290% water, ~4.8% lime	100 °C, 5 min in a steam kettle (in a perforated nylon bag)	15 h	Wash with water	Baking in a gas-fired oven with three moving tiers (further details not provided)	FB1	8790	Nixtamal, unwashed: ~−76|~−97 Nixtamal, washed: ~−73|~−96 Nejayote: n.d.|slight accum. Washing water: n.d.|slight accum. Masa: ~−87|~−92 Tortillas: ~−88|~−92 Tortilla chips: n.a.|~−94	Overall: Data of two production runs are given; tortilla chips were produced in only one run by frying in oil at 190 °C for 60 s. Of the initial FB1, a total of 62%–90% was recovered as FB1 or HFB1 ^e^ from the nejayote, mostly as HFB1	[46]
							HFB1	Probably n.d.	(Slight) accum. in all intermediate products and (by)products, highest accum. in nejayote		
							FB1 + HFB1	~12,000 nmol/kg	Nixtamal, unwashed: ~−48|~−85 ^f^ Nixtamal, washed: ~−48|~−87 ^f^ Masa: ~−70|~−78 ^f^ Tortillas: ~−72|~−82 ^f^ Tortilla chips: n.a.|~−86 ^f^		
							FB2	1,970	Nixtamal, unwashed: ~−32|~−97 Nixtamal, washed: ~−21|~−92 Nejayote: n.d.|n.d. Washing water: n.d.|n.d. Masa: ~−78|~−94 Tortillas: ~−71|~−91 Tortilla chips: n.a.|~−90	-	
19	Processing in a pilot plant according to commercial procedures including alkaline cooking, steeping, and washing of maize kernels, as well as baking and deep frying of masa to produce tortilla chips (details not provided)						FB1	220–46,500	Nejayote: slight accum. Masa: −88 to −94 ^d^ Baked tortilla chips: −76 to −90 ^d^ Fried tortilla chips: −36 to −78 ^d^	Ca. 34% and 45% of the initial FB1 ^f^ was detected as FB1, PHFB1, or HFB1 in the masa and nejayote, respectively. No indications for significant fumonisin–sugar adduct formation/accumulation	[45]
							PHFB1	n.d.−1340	Nejayote: n.d. or slight accum. Masa: ~−33 ^d^ to slight accum.		
							HFB1	n.d.−950	Nejayote: accum. Masa: no change to slight accum.		
							FB2	(Not provided)	Masa: ca. −89 to −94 ^d^ Baked tortilla chips: ca. −89 to −94 ^d^	-	
-	-	-	-	-	-	Baking in a tortilla oven at 260 °C, 20 s	FB1 + FB2	100–281	Tortilla chips: ‘as is’: −32 to −78 (mean: −59)	Use of maize flour mixtures. Commercial processing. Tortilla chips were prepared by frying tortilla strips at 170–175 °C for 40 s	[49]
20	~1% lime solution	~77% water, ~0.8% lime	7 min	18 h	No information	250 °C	FB1	1001	Cooked maize: −56 ^c^ Nixtamal: −83 ^c^ Masa: −80 ^c^ Tortillas: −83 ^c^	Commercial processing. Cooked maize was sampled before steeping	[50]
21	~0.7% lime solution	~200% water, ~1.5% lime	150 min	16 h	No information	250 °C	FB1	681	Cooked maize: −73 ^c^ Nixtamal: −80 ^c^ Masa: −85 ^c^ Tortillas: −89 ^c^		
22	~0.56% lime solution	~160% water, ~0.9% lime	10 min	16 h	No information	375 °C	FB1	1441	Cooked maize: −83 ^c^ Nixtamal: −88 ^c^ Masa: −100 ^c^ Tortillas: −100 ^c^		
23	~1.1% lime solution	~70% water, ~0.8% lime	120 min	18 h	No information	232 °C	FB1	1653	Cooked maize: −78 ^c^ Nixtamal: −88 ^c^ Masa: −100 ^c^ Tortillas: −100 ^c^		
24	~0.25% lime solution	200% water; ~0.5% lime	60 min	18 h	Rinse in 430% water (based on orig. maize mass)	Baking over an iron grill at 190–200 °C for ca. 4 min	FB1	150–11,800 (inoculated)	Masa: −46 to −99 ^c^ Tortillas: −26 to −98 ^c^	Higher reduction at higher initial FB1 level	
-	~0.25–1.6% lime solution	200% water; ~0.5–3.2% lime	15 or 60 min	18 h	As described above	As described above	FB1	150–11,800 (inoculated)	Masa/Tortillas: up to −100 ^c^	Higher reduction at higher lime concentration (and at higher initial FB1 level)	
25	0.33 or 1.67% lime solution	300% distilled water, 1% or 5% lime	90 °C, 15–60 min	17 h	2 rinses with 200% tap water (based on orig. maize mass)	-	FB1 + FB2	6,480–8,930	Masa: −26 to −48 ^c^	Of the initial FB1 + FB2^f^, a total of 64%–86% retained as parent form, PHFB, or HFB in the masa	[51]
							PHFB1 + PHFB2	110–260	Masa: no change to accum.	Increase at higher lime concentration	
							HFB1 + HFB2	n.d.	Masa: accum.	Higher accum. at higher lime concentration	
-	Distilled water, only	300% distilled water	As described above	As described above	As described above	-	FB1 + FB2	5230–20,380	Maize dough: −45 to −78 ^c^	Here, a total of 21%–55% of the initial FB1 + FB2^f^ retained as parent form, PHFB, or HFB in the maize dough	
							PHFB1 + PHFB2	140–510	Maize dough: −29 to −75 ^c^		
							HFB1 + HFB2	n.d.	Maize dough: n.d.		
26	0.5% Ca(OH)_2_ solution	100% water, 0.5% Ca(OH)_2_	Microwave (1650 W, 2450 Hz), 3.75 min	25 °C, 3.5 h; no removal of water indicated	-	270 °C, in total 54–55 s on each side	FB1 + FB2 + FB3	2137 (inoculated)	Masa: −6 (n.s.) ^c^ Tortillas: −54 ^c^	Use of maize grits	[48]
27	1.2% lime solution	Not specified	95–100 °C, 55 min	14 h	Wash with ca. 300% tap water	-	FB1	239,000 (inoculated)	Nixtamal: −83 ^c^ Nejayote: slight accum.	Pericarp removal during washing was avoided	[57]
							HFB1	n.d.	Nixtamal: accum. Nejayote: accum.		
							FB1 + HFB1^e^	239,000	Nixtamal: −39 ^c^ Nejayote: accum.		
28	~1.3% lime solution	325% water, ~4.1% lime	ca. 105 min	15 h	3 rinses with 275% water (based on orig. maize mass)	170–212 °C, ca. 3.5 min	FB1 + HFB1 ^e^	ca. 38,100	Tortillas: −46 ^f^	-	[47]
29	1.2% Ca(OH)_2_ solution	750% water, 9% lime	90–100 °C, 60 min	O/N	3 rinses with 750% water (based on orig. maize mass)	-	FB1	~23,314	Nixtamal: ~−90 ^c^	Of the initial FB1 ^f^, a total of ~47% retained as FB1, HFB1, or bound (H)FB1 in the nixtamal	[56]
							HFB1	~329	Nixtamal: ca. +1400 ^c^		
							Protein- bound (H)FB1	~69 (recovered as HFB1)	Nixtamal: ~+476 ^c^		
							Total bound (H)FB1	~89 (recovered as HFB1)	Nixtamal: ~+673 ^c^		
-	Water, only	750% water	As described above	As described above	As described above	-	FB1	~23,314	‘Mock-nixtamal’: ~−53 (n.s.) ^c^	Here, a total of ~44% of the initial FB1^f^ retained as FB1, HFB1, or bound (H)FB1 in the ‘mock-nixtamal’	
							HFB1	~329	‘Mock-nixtamal’: ~−60 (n.s.) ^c^		
							Protein- bound (H)FB1	~69 (recovered as HFB1)	‘Mock-nixtamal’: no change ^c^		
							Total bound (H)FB1	~89 (recovered as HFB1)	‘Mock-nixtamal’: ~+9 (n.s.) ^c^		
30	1.2% Ca(OH)_2_ solution	1,200% water, 14.4% lime	90–100 °C, 60 min	O/N	3 washes with 1200% distilled water (based on orig. maize mass)	-	FB1	9080 (inoculated)	Nixtamal: −77	Raw material: ground maize used as fungal growth medium. Nixtamal was prepared for a feeding trial. Concentrations and changes are given for the mixed diet	[62]
							HFB1	250	Nixtamal: ~+408		
							FB1 + HFB1	13,200 nmol/kg	Nixtamal: ~−54 ^f^		
-	Water, only	1,200% water	As described above	As described above	As described above	-	FB1	9080 (inoculated)	‘Mock-nixtamal’: −87		
							HFB1	250	‘Mock-nixtamal’: ~+120		
							FB1 + HFB1	13,200 nmol/kg	‘Mock-nixtamal’: ~−77 ^f^		
31	1.2% Ca(OH)_2_ solution	300% water, 3.6% lime	80–100 °C, 60 min	O/N	One wash with ca. 300% water (based on orig. maize mass)	-	FB1	45,200–48,000 (inoculated)	Nixtamal: ~−98 to −100	Nixtamal was prepared for a feeding trial. Concentrations and changes are given for the mixed diet	[64]
							HFB1	n.d.	Nixtamal: accum.		
							FB1 + HFB1	~62,600–66,500 nmol/kg	Nixtamal: ~−58 to −70 ^f^		

^a^: If not mentioned otherwise, maize was naturally contaminated. ^b^: Unless indicated otherwise, the change in the mycotoxin concentration is corrected for change in moisture content. Changes on a product ‘as is’ (wet weight) basis are indicated. Negative values: reduction; positive values: increase. ^c^: Here, change in the mycotoxin concentration is supposed to be corrected for change in moisture. ^d^: Here, it is not clear if the change in the mycotoxin concentration is corrected for change in moisture. ^e^: As equivalent to parent form. ^f^: On molar basis. ~: Approximate values that were calculated for this overview by using the data provided in the cited literature. |: Here, individual data of two production runs are given and separated by this symbol. FB1/2: fumonisin B1/2; HFB1/2: hydrolyzed fumonisin B1/2; PHFB1/2: partially hydrolyzed FB1/2. accum.: accumulation; n.a.: not analyzed; n.d.: not detected; n.s.: not significant; O/N: overnight; orig.: original.

**Table 3:** Effect of alkaline cooking (nixtamalization) of maize kernels and of entire tortilla production on contents of mycotoxins other than aflatoxins and fumonisins.

Study No. in Figure 2		Nixtamalization				Tortilla Baking on Hot Plate	Myco- toxin(s)	Initial Level in Raw Maize (µg/kg) ^a^	Corrected Change (%) ^b^	Comment(s)	Reference
	Alkaline Solution	Additions on Orig. Maize Mass Basis	Cooking	Steeping	Washing of Nixtamal						
32	2% Ca(OH)_2_ solution	Not specified	5 min (incl. stirring)	12 h	Thorough rinse with distilled water	110–120 °C, 7–8 min on each side	ZEN	230|4,230	Nejayote: n.d.|weak accum. Tortillas: −100|−59	-	[65]
							DON	3,280|12,260	Nejayote: n.d.|n.d. Tortillas: −82|−72		
							15-acetyl-DON	1,490|9,830	Nejayote: n.d.|n.d. Tortillas: −100|−100		
							ZEN	750|3,620 (spiked)	Nejayote: n.d.|weak accum. Tortillas: −71|−74	Mycotoxins were injected into the maize embryos	
							DON	850|4460|8250 (spiked)	Nejayote: n.d.|n.d.|n.d. Tortillas: −82|−72|–74		
-	-	-	-	-	-	Baking in a tortilla oven at 260 °C, 20 s	ZEN	4.5–8.7|19–24	Tortilla chips: ‘as is’: +116 to −7 (mean: +32)|−35 to −64 (mean: −49)	Use of maize flour mixtures. Commercial processing. Tortilla chips were prepared by frying tortilla	[49]
							DON	47–466	Tortilla chips: ‘as is’: +28 to −76 (mean: −32)		
33	0.25% lime solution	400% water, 1% lime	88 °C, 20 min	16 h	Two rinses with 250% water (based on orig. maize mass)	Baking in a gas-fired oven at ca. 365 °C for ca. 3 min	MON	1420	Cooked maize: −97 ^c^ Nixtamal: −100 ^c^ Nejayote: n.d. Wash water: n.d. Masa: −100 ^c^ Tortillas: −100 ^c^	Pilot-scale process	[68]
34	0.25% lime solution	400% water, 1% lime	88 °C, 20 min	16 h	Two rinses with 125% water (based on orig. maize mass)	ca. 250 °C, 3 min on each side	MON	17,640 (inoculated)	Cooked maize: −54 ^c^ Nixtamal (before washing): −64 ^c^ Nixtamal (washed): ~−69 ^c^ Nejayote: n.d. Wash water: n.d. Masa: ~−71 ^c^ Tortillas: ~−70 ^c^	Laboratory-scale	

^a^: If not mentioned otherwise, maize was naturally contaminated. ^b^: Unless indicated otherwise, the change in the mycotoxin concentration is corrected for change in moisture content. Changes on a product ‘as is’ (wet weight) basis are indicated. Negative values: reduction; positive values: increase. ^c^: Here, it is not clear if the change in the mycotoxin concentration is corrected for change in moisture. |: Here, data of different batches are separated by this symbol. ~: Approximate values that were calculated for this overview by using the data provided in the cited literature. DON: deoxynivalenol, MON: moniliformin; ZEN: zearalenone. accum.: accumulation; n.d.: not detected; orig.: original.
